# *Treponema pallidum*’s neural invasion: from blood-brain barrier breach to immune sabotage

**DOI:** 10.1128/iai.00621-25

**Published:** 2026-01-21

**Authors:** Han Yu, Sisi Zhao, Ke Yang, Ke Gao, Ting Lin, Peng Ling, Dingfa Deng, Feijun Zhao

**Affiliations:** 1 MOE Key Lab of Rare Pediatric Diseases & Institute of Pathogenic Biology and Key Laboratory of Special Pathogen Prevention and Control of Hunan Province, Hengyang Medical College, University of South China 34706, Hengyang, P.R. China; 2 Department of Critical Care Medicine, Affiliated Shaoyang Hospital of University of South China 37054, Shaoyang, P.R. China; 3 Department of Clinical Laboratory Medicine, Changsha Central Hospital 610468, Changsha, P.R. China; University of California Merced, Merced, California, USA

**Keywords:** *Treponema pallidum*, neurosyphilis, pathogenesis, central nervous system, immune response

## Abstract

Neurosyphilis is an infectious disease of the nervous system caused by *Treponema pallidum*. With the resurgence of syphilis worldwide, neurosyphilis has become prevalent again, but research on its pathogenesis remains challenging. *T. pallidum* exhibits remarkable invasive potential and immune evasion properties, which enable it to rapidly penetrate the blood-brain barrier (BBB) and infiltrate the central nervous system. Meanwhile, the immune response induced by this pathogen may cause tissue damage and accelerate disease progression. Additionally, host factors and the genotypes of *T. pallidum* strains are associated with susceptibility to neurosyphilis. This review systematically summarizes the latest literature on neurosyphilis, outlines recent advances in research on the effects of *T. pallidum* on the BBB, its immune interactions with the host, and omics-related studies, and aims to provide directions for future research on the pathogenesis of neurosyphilis.

## INTRODUCTION

Over the past several decades, the prevalence of primary and secondary syphilis has increased significantly. According to World Health Organization estimates, the number of global syphilis cases rose from 45 million in 1990 to 70 million in 2021 (1). Incident cases among individuals aged 15–49 increased from 7.1 million in 2020 to 8 million in 2022 (2). From 2019 to 2023, the total number of syphilis cases in the USA increased by 61%, reaching 210,000 cases (3). The incidence of syphilis is increasing, resulting in a rise in neurosyphilis patients.

However, there are few population-based studies currently available to estimate neurosyphilis prevalence, impeding the precise grasp of its actual epidemiological status. A study indicates that approximately 30% of untreated early-stage syphilis patients show the presence of *T. pallidum* in their cerebrospinal fluid (CSF), while the patients often have no symptoms. Four to ten percent of patients may progress to develop neurosyphilis (4). Another clinical study, based on CSF findings, reports that 3.5% to 8.0% of syphilis patients with clinical or ocular manifestations are diagnosed with neurosyphilis (5). Although neurosyphilis is less prevalent now than in the pre-penicillin era (6), the recent misuse of antibiotics, which is defined as irregular use deviating from clinical guidelines, has led to incomplete treatments. Such inadequate treatment contributes to a rise in chronic and latent syphilis, which in turn facilitates the development of neurosyphilis. Furthermore, treatment failure may occur due to the tolerance phenotype of *T. pallidum* or poor drug penetration into the central nervous system (CNS), leading to repeated episodes of neurosyphilis (7). As syphilis re-emerges, the incidence risk of neurosyphilis may have exceeded current reported levels. This condition not only challenges public health systems but also emphasizes the urgency of exploring its epidemiological drivers in depth and improving prevention and control strategies.

Neurosyphilis is classified as asymptomatic or symptomatic and further divided into early stages (presenting 1–2 years post-initial infection) and late stages (8). Notably, neurosyphilis may develop at any point during the syphilis infection (9). In the early phases of neurosyphilis, the most common manifestation is asymptomatic meningitis, which can only be diagnosed by detecting abnormalities in the CSF. If left untreated, as the disease progresses, neurosyphilis can involve the spinal cord or the brain’s meninges and parenchyma (10). Patients may experience cranial nerve involvement, ocular symptoms, as well as even suffer from a stroke. According to the clinical characteristics, neurosyphilis can be categorized into several types: asymptomatic neurosyphilis, meningeal syphilis, meningeal vascular syphilis, and more advanced forms known as substantive neurosyphilis—these include conditions such as paralysis dementia, tabes dorsalis, and ocular syphilis.

Routine neurosyphilis testing in the lab typically relies on reactive serological tests and CSF abnormalities (11). Patients typically exhibit increased white blood cell counts and modestly higher protein concentrations in the CSF. A positive result from the Cerebrospinal Fluid-Venereal Disease Research Laboratory (CSF-VDRL) test can confirm that patients with neurological symptoms have neurosyphilis and is generally regarded as the gold standard for its diagnosis. Nonetheless, this assay may exhibit limited sensitivity (below 30%) in the CSF of symptomatic individuals. Additionally, the CSF-VDRL test may remain reactive in asymptomatic neurosyphilis and long after adequate treatment (12).

With the global burden of syphilis-related neurological complications rising, elucidating neurosyphilis pathogenesis and refining its diagnostic and therapeutic processes accordingly hold practical value for advancing clinical standards. This review summarizes the latest research advancements regarding the pathogenesis of neurosyphilis, concentrating on how *T. pallidum* invades the CNS, the immune response it triggers, and the factors influencing susceptibility to infection.

## DISRUPTION OF THE INTEGRITY OF THE ENDOTHELIAL BARRIER

*T. pallidum* exhibits high invasiveness (13). It can infiltrate the body either through abrasions in the skin or by directly penetrating the mucosal membranes. During initial colonization, the pathogen adheres to both epithelial cells and components of the extracellular matrix (ECM) (14). Upon penetration of the epithelium, rapid local proliferation occurs, enabling dissemination through lymphatic and hematogenous pathways. Vascular endothelial cells constitute a key step in mediating the initial infection by *T. pallidum*. The pathogen’s interaction with host cells enables it to breach the endothelial barrier, thereby facilitating its hematogenous dissemination to diverse tissues and organs.

*T. pallidum* alters vascular permeability and induces vascular inflammation and tissue damage, thereby crossing the vascular endothelial barrier (15). The flexuous, flat-wave morphology of *T. pallidum* facilitates its penetration of vascular endothelial barriers, and its periplasmic motility structure drives movement via undulations along its length (14). When *T. pallidum* attaches to the inner walls of capillaries, it will break down the mucopolysaccharide material. This destruction affects capillaries that are rich in mucopolysaccharides, ultimately causing the vessels to collapse (16). In addition, mucopolysaccharide and sialic acid prevent antibodies binding, inhibit the activation of complement, and help the bacterium evade phagocytosis (17). *T. pallidum* may also bind to acidic mucopolysaccharides on cultured cells via mucopolysaccharidase on the organism’s surface (18). Furthermore, the hyaluronidase of *T. pallidum* acts as a spreading factor to promote bacterial spread to other tissues by directly interacting with and enzymatically degrading host hyaluronic acid (17). Research indicates that *T. pallidum*'s migration and penetration into endothelial cells may occur through paracellular pathways and cholesterol-dependent processes (19). Another study shows that *T. pallidum* promotes the degradation of the fibronectin matrix surrounding microvascular endothelial cells by activating the pAKT1/pSer39-vimentin signaling pathway, which enhances its ability to penetrate the endothelial barrier (20). The high vascular invasiveness of *T. pallidum* enables rapid spread within the host.

The outer membrane of *T. pallidum* is weak and devoid of lipopolysaccharides (LPS) typical of traditional gram-negative bacteria. However, it can produce various lipoproteins that interact with endothelial cells via membrane proteins, which disrupt the endothelial barrier and facilitate invasion of the host. Due to the limited advancements in *T. pallidum*’s *in vitro* culture, most research on *T. pallidum* has examined the interactions between its membrane proteins and host cells (21–23). For example, *T. pallidum* protein Tp0136 can change vascular permeability and stimulate angiogenesis via the PI3K/AKT pathway, which accelerates the spread of *T. pallidum* in the body (24). The progress of research on the interaction between *T. pallidum* membrane proteins and endothelial cells is illustrated in Table 1. However, some proteins are exposed and interact with the host only after the spirochete lyses. Therefore, the results of related *in vitro* experiments require further validation.

**TABLE 1:** *T. pallidum* membrane proteins interact with vascular endothelial cells^*a*^

Mycoprotein	Localization	Effects	Effectors	Mechanism/pathway	References
Tp47 (*tp0574*)	Periplasm	Increase in endothelial permeability	MCP-1, ICAM-1, MMP-1	ERK1/2-NF-κB, ROS	(25–28)
Tp17 (*tp0435*)	Periplasm	Disruption of endothelial cell-to-cell connections and endothelial adhesion	MCP-1, ICAM-1, E-selectin	–^*b*^	(29)
Tp0965	Outer membrane	Increase in endothelial permeability	ICAM-1, E-selectin, MMP-2	MAPK	(30, 31)
Tp92 (*tp0326*)	Outer membrane	Damage to endothelial cells	TNF-α, ICAM-1, IL-1β, IL-6, IL-8	NLRP3/caspase-1, chemerin/CMKLR1, MyD88/NF-κB	(32, 33)
TpF1 (*tp1038*)	Periplasm	Damage to endothelial cells	IL-8	CREB/NF-κB	(34)
Tp0751	Periplasm/outer membrane (debated)	Damage to endothelial cells	TNF-α, IL-1β, IL-6	Caspase 8/caspase 3	(35)
		Endothelial adhesion	Bind laminin, VE-cadherin, ECM protein, laminin receptor	–	(36–38)
Tp0155	Periplasm	Endothelial adhesion	Bind fibronectin	–	(39)
Tp0483	Periplasm	Endothelial adhesion	Bind fibronectin	–	(40)
Tp0750	Inner membrane	Endothelial adhesion	Bind laminin, MMP	–	(41)
Tp0136	Outer membrane	Endothelial adhesion and increase in endothelial permeability	Bind fibronectin and laminin, MMP-1, CCL-2, CSRP1, MYO10	PI3K-AKT, MAPK, NF-κB, RGD/integrin β1	(24, 42–44)

^*a*^ ROS, reactive oxygen species.

^*b*^ –, no mechanism/pathway has been confirmed to be associated with this protein to date.

## PENETRATION OF THE BLOOD-BRAIN BARRIER

After *T. pallidum* crosses the vascular endothelium barrier and enters the host’s bloodstream, it can rapidly invade the CNS. Experiments have demonstrated that *T. pallidum* can be detected in the CSF of rabbits a few days after exposure to the bacterium (45, 46). Five to forty percent of untreated individuals with primary, secondary, or early latent syphilis show *T. pallidum* presence in their CSF (47). These studies showed that *T. pallidum* exhibits early neuroinvasive capacity.

The blood-brain barrier (BBB) is the primary barrier to *T. pallidum*’s entry into the CNS. The blood plasma and brain cells are separated by a barrier, which is made up of the brain capillary wall—a collection of brain vascular endothelial cells and the basement membrane—and the choroid plexus. Brain vascular endothelial cells are essential for the barrier functions of the BBB (48).

Studies have indicated that *T. pallidum* can stimulate brain microvascular endothelial cells (BMECs). This stimulation leads to a reduction in ECM regulatory protein expression and structural changes, which may create conditions favorable for the dissemination of *T. pallidum*. Electron microscopy scanning demonstrates the attachment of *T. pallidum* to the brain microvascular endothelial cell membranes *in vitro* (49). The adhesin protein from *T. pallidum* is essential to this process, exemplified by the interaction of Tp0751 with BMECs through the laminin receptor (36). Some studies suggested that *T. pallidum* modulates the genes responsible for proteins that mediate bacterial adherence, endothelial cell stimulation, and the immune reaction in BMECs (50). The study of human brain microvascular endothelial cells (HCMEC/D3) demonstrated that *T. pallidum* stimulates IL-1β production, enhancing the expression of ADAMTS5 and increasing barrier permeability (35).

Apart from the cellular pathway, *T. pallidum* can cross the BBB via the paracellular pathway without disrupting its integrity (51). *T. pallidum* can traverse endothelial barriers and disrupt the architecture of the main endothelial junctional protein VE-cadherin by using a cholesterol-dependent, lipid raft-mediated endocytosis mechanism (38). This mechanism functions similarly to the interaction between white blood cells and invasive bacteria. Approximately a quarter of the body’s total cholesterol is found in the brain, primarily produced by astrocytes, a type of glial cell crucial for supporting neuron activity and maintaining synaptic linkages (52). This trait facilitates the penetration of *T. pallidum* into the CNS. Furthermore, *T. pallidum* protein Tp0751 triggers apoptosis and prompts the emission of the inflammatory cytokine IL-6, which may influence the expression of tight junction proteins (35).

The infection process of syphilis involves opsonized phagocytosis, non-opsonic phagocytosis, and active invasion in the interaction between *T. pallidum* and macrophages (53). Protozoa can use the “Trojan horse” mechanism to hide inside cells, cross the BBB, and enter the CNS (54). Whether *T. pallidum* adopts a similar mechanism remains inconclusive and requires further research for verification.

**Fig 1:**
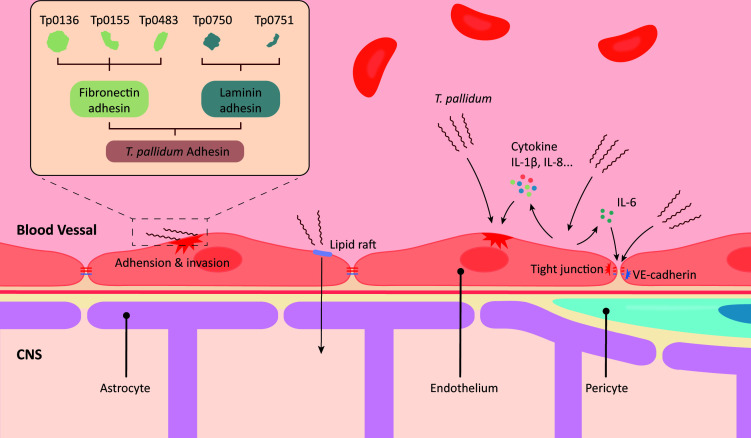
The mechanism of *Treponema pallidum* crossing the blood-brain barrier. *T. pallidum* attaches to the surface of endothelial cells through its motility, chemotaxis, and adhesion proteins. Subsequently, *T. pallidum* penetrates the blood-brain barrier by disrupting endothelial cells and junctions or by using paracellular pathways.

The aforementioned studies have revealed key clues about *T. pallidum*’s penetration of the BBB by barrier structure, molecular interactions, and cellular functions (Fig. 1). Furthermore, speculations about the Trojan horse mechanism have opened a new path to explore its complete neuroinvasion mechanism.

## IMMUNE RESPONSE AGAINST *T. PALLIDUM* INFECTION

A key reason for syphilis’ rapid spread is *T. pallidum*’s effective evasion of host immune attacks (55). This allows it to persist in the host over an extended period, culminating in the development of neurosyphilis. The clearance of *T. pallidum* and the occurrence and progression of syphilis are highly associated with the host’s immune response. *T. pallidum* can further enhance immune evasion by affecting host immune cells, ultimately leading to chronic persistent infection.

**Fig 2:**
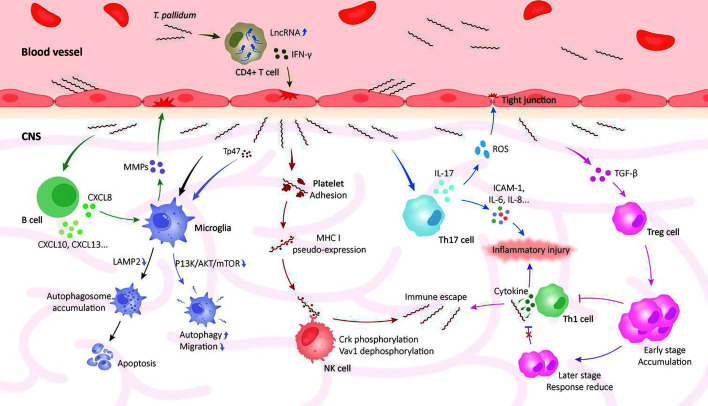
Interaction between *Treponema pallidum* and the host immune system. In neurosyphilis, *T. pallidum* initiates an immune disturbance and a massive inflammatory response that leads to damage to host tissue but does not cause substantial damage to neurons. *T. pallidum* stimulates immune cells to release various cytokines. The release of these cytokines disrupts the host’s immune system, leading to inflammatory damage and immune evasion.

A notable pathological characteristic of neurosyphilis is a robust and compartmentalized neuroimmune response within the CNS, without direct neuronal damage (56). In addition, recent studies show that inflammatory cells in peripheral blood cause neurological damage by disrupting the BBB in individuals with neurosyphilis. The brain tissues of neurosyphilis patients revealed marked activation of both humoral and cellular immunity, along with perivascular infiltration by diverse inflammatory cells (57). These findings reflect the complex role of immune responses in the pathological process of neurosyphilis (Fig. 2).

## INNATE IMMUNITY

Microglia, natural killer (NK) cells, and various molecules participate in the innate immune response to eliminate *T. pallidum* in early syphilis after it invades the skin barrier (58).

The activation of microglia in the CNS is extremely significant during neurosyphilis (59). Microglia are immune cells residing in the CNS that act as the main defenders, crucial for neuroinflammation and immune monitoring. *T. pallidum* is significantly toxic to microglia, mainly by disrupting their autophagy. Autophagy serves a dual purpose in neuroinflammation: it protects cells from damage, yet when dysregulated, it may cause cell death. *T. pallidum* can inhibit TFEB-mediated lysosomal biogenesis in microglia by activating the mTORC1 signaling pathway (60). Alternatively, it may suppress the expression of hexokinase 2, which affects the glycolytic pathway (61). This inhibition hampers the production of lysosomal-associated membrane protein 2 and disrupts the fusion of autophagosome-lysosome, thereby blocking autophagic flux. Additionally, *T. pallidum* flagellin FlaB3 enhances inflammatory responses in HMC3 cells by inhibiting autophagy (62). Several studies also show that inhibition of the autophagic flux and accumulation of autophagosomes lead to apoptosis (63–65). Studies conducted *in vitro* suggest that autophagy may also modulate the neuroinflammatory response in microglia following LPS stimulation, potentially influencing associated neurotoxicity (66, 67). This process prevents microglia from clearing *T. pallidum*, resulting in immune evasion.

The membrane protein Tp47 of *T. pallidum* is essential for its interaction with microglia. Tp47 is found in high levels in the CSF of neurosyphilis patients (68). Tp47 triggers autophagy and cell death through the PI3K/AKT/mTOR pathway and inhibits microglial migration (69). Furthermore, Tp47 activates autophagy via two primary routes associated with endoplasmic reticulum stress: PERK/ATF4 and IRE1/XBP1, which enhance the phagocytic activity of HMC3 cells (70).

NK cells are essential components of the first line of defense against diverse microbial pathogens, such as viruses and intracellular bacteria (71). A study revealed a general decline in the percentage of NK cells in the peripheral blood samples from individuals with neurosyphilis. This decline indicates that the progression of disease may be linked to the loss of NK cells (72). *T. pallidum* adhered to and stimulated platelets, encouraging the release of particles. This process results in platelets expressing and releasing abundant MHC class I molecules, which are then transferred to the surface of *T. pallidum*. This transfer could trigger an immune response marked by the “pseudo-expression” of MHC class I on *T. pallidum*’s surface (73). MHC class I interacts with the KIR2DL3 receptor on NK cells, leading to Vav1 dephosphorylation and Crk phosphorylation (74). *In vitro* experiments involving NK cells and *T. pallidum* suggest that the pathogens can likely evade recognition and killing by NK cells.

## T CELL-MEDIATED RESPONSE

CD4+ T cells are the primary immune cells reacting to *T. pallidum* (75). In a model of invasion of the CNS by the non-human primate *T. pallidum*, it was found that the number of T cells increased over time when compared to B cells (76). Furthermore, there was an increase in the proportion of CD4+ T cells relative to CD8+ T cells. *T. pallidum*-specific CD4+ T cells can be detected in the blood and skin of syphilis patients during their initial visit, and they can persist for up to 10 years after treatment (77).

Th17 cells represent a subgroup within CD4+ T helper cells. Patients with neurosyphilis exhibit markedly elevated levels of Th17 cells in their CSF, with IL-17 levels progressively increasing as the disease advances (78). IL-27 levels in CSF are negatively correlated with CSF-VDRL titers. IL-27 inhibits the differentiation of Th17 cells and inflammatory responses, playing a key role in both adaptive and innate immunity. In contrast, IL-17 influences the clinical symptoms of patients (79). IL-17 is a potent pro-inflammatory cytokine produced by Th17 cells. It enhances the synthesis of IL-6, IL-8, and ICAM-1, which exacerbates local inflammation and results in inflammatory damage. In addition, IL-17A stimulates the production of reactive oxygen species via NADPH oxidase or xanthine oxidase pathways. The oxidative stress resulting from this activation triggered the endothelial contractile machinery, leading to an expansion of the intercellular gap in the endothelial cell monolayer. This expansion was accompanied by a downregulation of the tight junction molecule occludin (80). The destruction of intercellular tight junctions results in the impairment of the BBB’s protective function.

Increasing evidence suggests that Treg cells significantly contribute to the development of neurosyphilis (81). Treg cells, a CD4+ T-cell subset with immunosuppressive functions, secrete TGF-β and IL-10 to regulate the host’s immune reaction and prevent Th1-mediated immunoreaction (82). Treg cells and Th17 cells share developmental routes, and changes in their fate decisions influence anti-inflammatory and pro-inflammatory responses (83). Syphilis patients suffering from neurosyphilis exhibit notably higher counts of regulatory T cells in their peripheral blood than those without neurological symptoms. This correlation implies that the escalation of neurological symptoms in syphilis patients is tied to elevated levels of Treg cells in their bloodstream. Individuals with symptomatic neurosyphilis had significantly lower Treg cell levels, both in percentage and total number count, compared to those with asymptomatic neurosyphilis (84). The findings indicate that excessive Treg buildup in the CNS could hinder the control of T cell-induced inflammation and tissue harm in the brain and spinal cord, leading to neurological manifestations. Therefore, it is speculated that the overactivation of Treg responses in the early stages of infection favors the survival of *T. pallidum*. In contrast, during the later stages of the disease, reduced Treg responses do not prevent T cell-mediated tissue damage. This failure leads to the development of symptoms in neurosyphilis patients. Research has also shown that TpF1 protein downregulates the immune response, stimulates MΦ to produce and release anti-inflammatory cytokines, and promotes the development of Treg cells (85).

A clinical study shows that neurosyphilis patients had considerably more CD3+CD8+ lymphocytes than healthy controls. Additionally, their CD4+/CD8+ T-cell ratio was much lower than that of the healthy control group (72). The findings suggested a steady decline in CD3+CD8+ cell counts among asymptomatic, early, and late neurosyphilis patients. This indicates that persistent *T. pallidum* infections may exhaust CD3+CD8+ lymphocytes. Patients with symptomatic neurosyphilis show elevated CD8+IFN-γ+ and reduced CD8+IL-17+ cell counts. In addition, in patients without HIV infection, CD8+IFN-γ+ cells may be a more accurate indicator to differentiate for distinguishing neurosyphilis from non-neurosyphilis syphilis (86). These offer novel perspectives on the pathophysiological mechanisms of symptomatic neurosyphilis.

lncRNA-ENST00000421645 levels in peripheral CD4+ T cells were markedly elevated in neurosyphilis cases versus non-neurosyphilis syphilis and control groups (87). This lncRNA sponging of PCM1 results in increased expression of IFN-γ and decreased inhibition of PKC (88). In alignment with this observation, individuals with neurosyphilis display elevated levels of IFN-γ in their CSF when contrasted with those without neurosyphilis (89). An excessive increase in IFN-γ may damage endothelial cells, result in the accumulation of inflammatory cells, and promote the occurrence and development of neurosyphilis.

## B CELL-MEDIATED RESPONSE

Emerging evidence has shown that B cells play crucial roles in the CNS pathological damage in neurosyphilis. Previous studies have shown that the levels of CD19+ B cells, memory B cells, and intrathecal immunoglobulin are notably elevated in CSF of neurosyphilis patients (90). This suggests that B cells are activated and aggregate within the CNS.

Analytical results from neurosyphilis patient CSF samples demonstrated a substantial increase in Chemokine C-X-C motif ligand 13 (CXCL13) concentrations (91). CXCL13, also referred to as BLC or BCA-1, exhibits high expression levels in the human liver, spleen, lymph nodes, and intestines (92). Overexpressed CXCL13 recruits B cells from the CSF to form an ectopic germinal center (EGC). When the protective mechanism against autoimmune responses caused by abnormal B-cell activation in the CNS is absent, EGCs can trigger abnormal humoral immune responses in the CNS (93). Typically, CXCL13 lacks direct access into the CSF via the bloodstream, even in cases of severe BBB dysfunction. Therefore, heightened concentrations of CXCL13 in the CSF may originate from the CNS (94). CSF CXCL13 concentrations and CXCL13 quotient {QCXCL13 = [(CXCL13_CSF_ / Albumin_CSF_) / (CXCL13_Serum_ / Albumin_Serum_)]} can also be used as a key indicator differentiating neurosyphilis from non-neurosyphilis syphilis (95). Furthermore, CXCL13 levels significantly decrease after treatment in neurosyphilis patients. This reduction correlates with improved patient outcomes, demonstrating its strong potential as a predictive marker for therapeutic efficacy (96).

These studies indicate that biomarkers associated with humoral immunity hold promise for use in both the diagnosis and prognostic evaluation of neurosyphilis. Identifying their potential value may offer new ideas for advanced research in this area.

## FACTORS AFFECTING SUSCEPTIBILITY TO NEUROSYPHILIS

Host factors influence the natural progression of syphilis, increasing the risk of neurosyphilis. A family of 10 proteins known as human toll-like receptors (TLRs) recognizes pathogen-associated molecular patterns in different ways. Single-nucleotide polymorphisms (SNPs) in TLR1 (1805 T→G), TLR2 (2258 G→A), and TLR6 (745 C→T) are associated with a higher risk of neurosyphilis (97).

Furthermore, the functional polymorphism of the IL-10 promoter is associated with neurosyphilis. Individuals with neurosyphilis exhibited a notably elevated frequency of IL-10 promoter −1082 GG and −592 CC SNP genotypes relative to those without the condition. Syphilis patients with these genotypes had higher levels of IL-10 in their CSF (98). Excessive production of IL-10 can inhibit pro-inflammatory response and promote neurological progression in syphilis. Increased IL-10 production may lead to persistent syphilis infection, which is crucial in the pathophysiology and progression of neurosyphilis (99). In particular, the concentration of IL-10 in CSF is helpful for the detection of neurosyphilis, particularly asymptomatic cases.

Numerous studies have found a certain correlation between the genotype of *T. pallidum* strains and the occurrence and development of neurosyphilis. In an animal experiment, researchers infected rabbits with three different strain types (14a/a, 14e/b, and 14d/f). They found that the neuroinvasion was most severe in 14a/a and 14d/f (100). Type 14d/f was the predominant strain among individuals with neurosyphilis. Therefore, compared with other strain types, type 14d/f exhibits heightened neuroinvasiveness and superior immune evasion capabilities within the CNS compared to other strains. In Shanghai, China, there is no substantial correlation between type 14d/f and neurosyphilis, while type 19D/C is associated with neurosyphilis (101). Nevertheless, the regional heterogeneity observed in these studies may be attributed to both the genetic diversity of endemic *T. pallidum* strains and disparities in clinical diagnostic criteria coupled with public health surveillance systems.

The occurrence and development of neurosyphilis also seem to be affected by co-infections. The patient populations affected by human immunodeficiency virus (HIV) and *T. pallidum* are similar, and co-infection is quite common (102). Although co-infection with HIV does not heighten the risk of developing neurosyphilis, compared to patients without HIV infection, those with co-infection may display neurological features earlier and may not respond fully to treatment (103).

Both host and pathogen factors influence the occurrence, development, and clinical outcome of neurosyphilis. With the advancement of molecular biology technologies, these factors offer novel insights to improve neurosyphilis risk assessment, diagnostic workflows, and therapeutic strategies.

## OMICS RESEARCH AND NEUROSYPHILIS

As omics research offers unique advantages in exploring disease mechanisms, it is gradually emerging as a popular research area. Through the comparison of metabolite differences between the infected and healthy cohorts, coupled with bioinformatics analysis, a panel of potential biomarkers that may indicate the pathological status of neurosyphilis can be identified.

Neurosyphilis patients show significant differences in N-acetyl-L-tyrosine, D-mannose, L-gulono-γ-lactone, and hypoxanthine when compared to both non-neurosyphilis and those without syphilis (104). L-Gulono-γ-lactone serves as a precursor of vitamin C. A decline in brain vitamin C levels can lead to harmful oxidative stress and cognitive abilities, particularly in inflammatory neurodegenerative diseases (105). This could indicate a possible link to neurosyphilis.

Another metabolomics study of CSF from patients with neurosyphilis, the tryptophan-kynurenine pathway exhibited significant alterations in neurosyphilis patients, along with observed changes in the levels of its metabolites, 5-hydroxy-L-tryptophan and acetyl-N-formyl-5-methoxykynurenamine (106). In addition, the concentrations of bilirubin and L-histidine increase in the CSF of neurosyphilis patients, while those of prostaglandin E2 and alpha-kamlolenic acid decrease (47). L-Histidine decarboxylates to produce histamines, which are known for their strong vasodilatory effects. Histamines are associated with various allergic reactions and inflammatory conditions (107), potentially leading to pathological damage in patients with neurosyphilis. The role of these differential metabolites in neurosyphilis development needs further investigation.

The CXC chemokine family received considerable attention in recent years. Significantly higher levels of chemokines CXCL6, CXCL7, CXCL8, CXCL10, and CXCL13 are observed in neurosyphilis patients versus uninfected individuals (108–110). The relationship between CXCL13 and B cells has been described in the preceding sections. CXCL8 binds to its receptors, CXCR1 and CXCR2. This interaction enhances the synthesis of matrix metalloproteinases in microglia, which leads to the rupture of the BBB and allows inflammatory cells to enter the brain (111). Although the CXC chemokine family is not specific, numerous studies suggest that it may be a potential direction for further exploration of the mechanisms of neurosyphilis.

Novel bioinformatics analysis methods have also provided a new perspective for the mechanistic research of neurosyphilis. A study integrated Mendelian randomization with transcriptomic data for comprehensive analysis, which identified METAP2 as a key biomarker of neurosyphilis and verified its potential utility (112).

The differential markers identified through CSF metabonomics provide new insights for future research on neurosyphilis, and elucidating the function of these metabolites in neurosyphilis pathogenesis warrants deeper investigation.

## CONCLUSIONS AND OUTLOOK

Neurosyphilis is an important complication of syphilis, and its pathogenesis has not been fully elucidated, posing significant challenges in clinical diagnosis and prevention. In this review, we present the latest research progress on the pathogenesis of neurosyphilis. Additionally, we discuss the role of immune responses in the pathogenesis of neurosyphilis and the factors influencing susceptibility to neurosyphilis, and analyze the potential connections between the metabolomics studies of CSF in neurosyphilis patients and the pathological features of neurosyphilis.

In recent years, reviews on neurosyphilis have mainly focused on the clinical diagnosis, symptoms, staging, and treatment of neurosyphilis. Owing to the distinctive immune evasion capabilities of *T. pallidum*, neurosyphilis pathogenesis research has always progressed slowly. Vaccination represents the most cost-effective intervention for infection control. However, syphilis vaccine development has been considerably hindered by the reliance on rabbit models for assessing recombinant protein immunization (113). Although a few studies have evaluated the feasibility of mice model simulating syphilis infection, it has not yet been fully validated and requires further research (114). Breakthroughs in *in vitro* cultivation of *T. pallidum* and development of reliable new animal models are particularly important for further exploring neurosyphilis mechanisms.

The structure and morphology of *T. pallidum* facilitate its passage through the BBB during the early stages of infection, with *T. pallidum* membrane proteins playing a crucial role in this process. Once in the CNS, *T. pallidum* triggers a series of immune responses, with CD4+ T cells being the primary responders to the spirochete. Notably, the immune response in neurosyphilis is intense, yet *T. pallidum* may not directly damage neurons. With advancements in research and technology, the CSF omics analysis of neurosyphilis has shown significant advantages in exploring mechanisms. Metabolomics can be employed to thoroughly examine the host’s reaction against *T. pallidum*, pinpointing critical biomarkers and therapeutic intervention targets, which provides new perspectives for studying its pathogenesis. Additionally, host gene polymorphisms also offer new ideas for exploring the pathogenesis of neurosyphilis. However, the current research on the pathogenesis of neurosyphilis mainly explores the changes in immune responses from cellular or clinical samples. The complex mechanisms of *T. pallidum*’s interaction with host cells remain incompletely understood. Screening more specific biomarkers from the identified metabolite alterations, such as METAP2 or lncRNA, and fully elucidating their role in mediating neuroinflammation could pave the way for significant breakthroughs in understanding neurosyphilis mechanisms.

In summary, research on neurosyphilis requires a deeper examination of the relationships between *T. pallidum* and the host, building upon current knowledge. Through a systematic summary of current studies, we hope to provide new perspectives for neurosyphilis research and establish a robust scientific base for the advancement of novel preventive, diagnostic, and therapeutic strategies.
